# Antibacterial Cellulose Nanocrystal-Incorporated Hydrogels With Satisfactory Vascularization for Enhancing Skin Regeneration

**DOI:** 10.3389/fbioe.2022.876936

**Published:** 2022-04-26

**Authors:** Haibin Lu, Xiaoling Li, Mu Zhang, Changpeng Xu, Wenqiang Li, Lei Wan

**Affiliations:** ^1^ Stomatological Hospital, Southern Medical University, Guangzhou, China; ^2^ Shunde Hospital, Southern Medical University (The First People’s Hospital of Shunde), Foshan, China; ^3^ Department of Orthopaedics, Guangdong Second Provincial General Hospital, Guangzhou, China; ^4^ Engineering Technology Research Center for Sports Assistive Devices of Guangdong, Guangzhou Sport University, Guangzhou, China

**Keywords:** hydrogel, cellulose nanocrystals, angiogenesis, antibacterial activity, skin regeneration

## Abstract

Wound healing of skin defects remains a significant clinical problem due to inflammation, infection, and dysangiogenesis; especially, the promotion of microvasculature formation in healing of chronic wound or deep skin defects is critical as it supplies oxygen and nutrients to the impaired tissue, relieving uncontrolled inflammatory responses. The cellulose nanocrystals (CNCs) in the liquid crystalline phase, which facilitates cell proliferation and migration, has been shown to improve vascularization effectively. Therefore, we developed a novel injectable hydrogel based on Schiff base and coordination of catechol and Ag. The obtained hydrogels (CCS/CCHO-Ag) exhibited *in situ* forming properties, satisfactory mechanical performance, controlled release of Ag, antibacterial capacity, and biocompatibility. In addition, the hydrogels could also entirely cover and firmly attach wounds with irregular shapes, so as to reduce the re-injury rate. More importantly, experiments *in vitro* and *in vivo* demonstrated that CCS/CCHO-Ag hydrogels can promote neovascularization and tissue regeneration, thanks to their anti-inflammatory and antibacterial effects. In conclusion, these multifunctional hydrogels are well on the way to becoming competitive biomedical dressings, which show tremendous potential application in the field of tissue engineering.

## Introduction

In severe cases, skin wounds can cause disability and even be life-threatening, which is a notable issue that deserves immediate attention (Lei et al., 2020; Li et al., 2020; Sheng et al., 2021). Currently, the treatment of chronic skin wounds is still a clinical challenge, and more than 50% of chronic wounds are refractory to current therapies (Li et al., 2020). Wound dressing, particularly hydrogel, plays an important part in skin repair and structure remodeling (Khan et al., 2020; Peng et al., 2020). Backed by hydrophilic three-dimensional networks, hydrogels create a breathable and moist environment in which wounds can heal more rapidly, suggesting that they are one of the most effective wound dressings (Majumder et al., 2020; Mao et al., 2020; Tamahkar et al., 2020). Due to excellent biocompatibility and biodegradability, polysaccharides such as hyaluronic acid and chitosan are the most commonly used natural biopolymers in the wound dressing field (Nosrati et al., 2021). For example, Cao et al. (2021) prepared a novel carboxymethyl chitosan hydrogel used for wound healing, which exhibited thermoresponsive capacity to accelerate wound closure and surrounding tissues regeneration. Yang et al. (2016) proposed a facile approach to produce a polyacrylamide–chitosan (PAM-CS) composite hydrogel with dramatically improved tensile strength and toughness, which can efficiently accelerate skin repair at different wound healing periods. Unfortunately, these preparation of hydrogel dressings show poor antibacterial performance and weak biological adhesion, thus greatly limiting their further application in the wound dressing market.

Generally, silver (Ag) nanoparticles have attracted much attention owing to distinctive chemical and physical performance, low toxicity, and broad-spectrum antibacterial ability (Xie et al., 2018; Basha et al., 2020). It was reported that Ag-loaded nanocomposite hydrogel can be used as a controlled release carrier of the bacteria-killing activity for accelerating wound healing (Yang et al., 2021), but the reported hydrogel system based on UV polymerization of the polymer matrix restricts their application in irregular skin wounds, thanks to their inherent traits that do not completely avoid infection. Recently, the development of injectable hydrogels with unique shape adaptability and self-healing ability contribute to complete fit and adequate contact between the wound and hydrogel dressing and thereby promote the therapeutic efficacy on the injuries, which has aroused considerable interest. Through non-covalent cross-links such as coordination bonds, hydrogen bonds, ionic bonds, and Schiff bases to form injectable self-healing hydrogels can be endowed with good adhesion behavior, mechanical strength, and biological activity (Guo et al., 2015; Huang et al., 2019; Liu et al., 2019). Moreover, the injectable self-healing hydrogel fabricated by dynamic cross-link show favorable potential for 3D bioprinting, which can also provide a 3D microenvironment for complex tissue regeneration (Kumar et al., 2020; Guyot et al., 2021). Nevertheless, long gelation time and low mechanical strength of hydrogel remain an important challenge to develop injectable self-healing hydrogels as 3D bioink. In wound dressing, another critical issue of the chronic wound or full-thickness skin defect is impaired angiogenesis that supplies oxygen and nutrients for cells and tissues (Balagangadharan et al., 2018; Chen et al., 2019a; Chen et al., 2019b). Exogenous angiogenic growth factors (GFs), such as vascular endothelial growth factor (VEGF), are usually exploited to facilitate wound closure and angiogenesis (Huang et al., 2019). Unfortunately, the instability of VEGF in a highly proteolytic and oxidative environment of the wound leads to large limitation of its clinical application.

Important biomolecules such as polypeptides, proteins, nucleic acids, lipids, and polysaccharides in organisms have liquid crystal states (Chen et al., 2019b). Previous studies have proved that the cell membrane has special conformation similar to the liquid crystalline (LC) state, and its surface, which is always in contact with blood, is in a flowing lipid LC condition (Jewell, 2011; Wu et al., 2017). Since this viscoelastic material can be seen as a soft elastic solid, LC has the potential to design the cellular surface and to study cell adhesion (Lockwood et al., 2006). The alignment structure of LC not only facilitates the ring formation of endothelial cells but also has positive effects on cell migration, leading to promote angiogenesis due to LC stimulation *via* tunable physical properties and anisotropic viscoelastic behavior (Zhan et al., 2021). Cellulose nanocrystals (CNCs) (from cellulose with a rod-like structure) have the ability to self-assemble from the liquid phase to the cholesteric LC phase (Lin et al., 2017; Chen et al., 2021). It is important to note that surface chemical modification makes the formation of injectable hydrogels available while ensuring high-water and biocompatible properties to apply to biomedicine. For example, Kumar et al. (2019) prepared carboxylated CNC-reinforced and ionically cross-linked polysaccharide hydrogels and further designed 3D printable carboxylated CNC-reinforced hydrogel inks for tissue engineering (Kumar et al., 2020). To date, there is almost no report on CNCs with the LC state and the promotion of vascularization during wound healing, which are expected to be promising LC biomaterials for regeneration of blood vessels as cells can sense and respond to the microenvironment constructed by the LC state and upregulated angiogenic factors (Zhan et al., 2021).

Inspired by the mussels’ high adhesion to various substrates, catechol has been introduced into the polymer matrix, which usually displays strong and long-term adhesiveness to soft tissues. More importantly, the catechol group can be coordinated with metal ions (including Mg^2+^, Fe^3+^, and Ag^+^) by facile mixing to enhance gelation of the hydrogel (Yuan et al., 2021). Here, we designed a newly multifunctional hydrogel with double cross-linking for wound healing. First, hydrocaffeic acid was used to graft the CS by the amidation reaction to prepare catechol-modified CS (CCS), which acts as the backbone of hydrogel, and aldehyde-modified cellulose nanocrystals (CCHOs) play roles as both physical fillers and cross-linkers in the fabrication of hydrogels. Then double cross-linking of Ag-catechol and Schiff base bonds are used as part of the gel-forming mechanism, and a bio-multifunctional hydrogel (CCS/CCHO-Ag) is produced by introducing Ag to catechol-modified chitosan (CCS) and aldehyde-modified cellulose nanocrystals (CCHOs). As a result, the obtained hydrogel system show enhanced mechanical behavior, good injectable properties, tissue adhesion, long-term antibacterial, and cytocompatibility. Based on these unique properties, a full-thickness skin wound model was created for investigating the wound healing efficiency of hydrogel dressings *in vivo*.

## Experimental Section

### Materials

Cellulose was supplied by Fujian Qingshan Paper Industry Co., Ltd., China. Chitosan (degree of deacetylation ≈95%, 100–200 mps), 3,4-dihydroxyhydrocinnamic acid, 1-ethyl-3-(3-dimethylaminopropyl)-carbodiimide hydrochloride (EDC), silver nitrate (AgNO_3_), and sodium borohydride (NaBH_4_) were all purchased from Aladdin Biochemical Technology Co., Ltd. (China). In terms of other analytical reagents, they were obtained from the Tianjin Damao Chemical Reagent Factory.

### Cellulose Nanocrystals

A certain amount of cellulose was pretreated with 5 wt% NaOH solutions at 70°C three times. The resultant specimen was further washed with DI water and dried. A measure of 10 g of dried cellulose was immersed in 100 ml of 64 wt% H_2_SO_4_ solution and heated to 65°C for 90 min A volume of 2L DI water was used to stop the reaction. The acid was removed by centrifugation, and the final suspension was dialyzed in a dialysis tube (MW 8000–14000) for a week. The concentration of the CNC was set at 2.5 wt%.

### Synthesis of Modified CNCs (CCHOs)

A measure of 50 g of CNC suspension was heated to 35°C and 2 g NaIO_4_ was added to it under vigorous stirring. The reaction was carried out in a dark room for 12 h and quenched with the addition of 15 ml glycol. The product was dialyzed in a dialysis tube (*M*_W_ 7500–14000) for 3 days to remove excess glycol and impure ions. The aldehyde group content of CCHOs was determined using a NaOH titration method, according to previous studies (Yin et al., 2016). The morphology and distribution of the CNCs and CCHOs were studied *via* a field emission scanning electron microscope (FE-SEM, ULTRA 55, Carl Zeiss, Germany) and transmission electron microscopy (TEM, Philips CM-120, Eindhoven, Netherlands). A polarizing optical microscope (POM, BX53M, Olympus, Tokyo, Japan) equipped with a digital camera (DP200) was used to study the optical phenomenon of the CNCs or CCHOs whisker aqueous suspensions with 0.5, 1, and 2% (w/v).

### Synthesis of Catechol-Modified Chitosan (CCS)

Based on the EDC activation reaction, CCS was produced by the esterification between the NH_2_ group (chitosan) and the carboxyl group (hydrocinnamic acid). In brief, chitosan was poured into 1 N HCl (aq) solution, and its pH was regulated by 1 N NaOH (aq) to pH of 5 under vigorous stirring. Then hydrocaffeic acid dissolved in DI water was added to the above solution. Subsequently, EDC dissolved in DI water and equal volume of ethanol solution (1:1, v/v) were also added. The pH of the mixed solution was adjusted to 5 and stirred constantly for 5 h. Finally, the obtained solution required dialysis to remove residual reagents after completion of all reaction steps, including 3 days in acidified DI water (pH = 5.0) and 8 h in DI water. CCS was obtained after lyophilization and observed under FTIR spectroscopy (Fts6000, Bio-Rad, United States). In addition, the resulting freeze-dried samples characterized by ^1^H NMR and UV-vis spectroscopy. The catechol substitute ratio of CCS was determined by ^1^H NMR (Bruker Biospin GmbH, Germany).

### Formation of CCS/CCHO-Ag Hydrogels

The resultant CCS (0.25 g) dissolved in 10 ml CCHO suspensions with a molar ratio of 0.5, 1.0, and 2.0 for -CHO/-NH_2_, respectively. Then 0.2 mM AgNO_3_ was added to the abovementioned solution, and the pH of the solution was adjusted above nine by NaOH after stirring for 30 s. Next, the pregel solution was incubated at 37°C for 5 min to trigger the transformation to a hydrogel through the Schiff base and the coordination bond. The gelation of CCS/CCHO-Ag was evaluated by the tube inverted test. Finally, the obtained hydrogels were washed with PBS solution three times for purification. The functional groups of the prepared hydrogels were tested by FTIR spectroscopy (Fts6000, Bio-Rad, United States).

### Characterization of Hydrogels

#### Rheological Measurements

The rheological behavior of the hydrogels was investigated using a rotational rheometer (DHR, TA Instruments, United States) at 37°C. The viscosity of the whisker aqueous suspensions was measured in the shear rate range from 0.1 to 10 s^−1^. Then the gelation time of the mixtures was investigated by time sweeps at a constant oscillatory strain of 1% and frequency of 6.28 rad/s. Also, the frequency sweep oscillatory tests were carried out by varying amplitude-frequency of 0.1–100 rad/s (a strain of 1%). At last, the strain sweep was performed with the oscillatory strain from 0.1 to 100% to make sure this modulus value was within the linear elastic range (a frequency of 6.28 rad/s).

#### Scanning Electron Microscope Observation

The morphology of resulting hydrogel specimens were detected using a scanning electron microscope (SEM, LEO1530 VP, Philips, Netherlands). First, the prepared hydrogels were transferred to a freezer with −80°C and dried by using a freeze dryer. After that, the freeze-dried samples were cryogenically fractured in liquid nitrogen condition. Then the cross sections of the hydrogel samples were treated by gold spraying (Sputter Coater, Ted Pella, LJ-16) for SEM observation, and the pore diameter was determined by ImageJ analysis.

#### Mechanical Behavior

All hydrogels were made cylindrical in shape with a diameter of 10 mm and a thickness of 8 mm for the following compression tests, which were conducted by a dynamometer machine (AGI-1, Shimadzu, 1 kN load, Japan), and the Young’s modulus values were calculated from the compression stress–strain curves of hydrogel between 40 and 60% strain. In addition, cycling compression tests were also performed with a speed of 2 mm/min and in the 0–70% range. In particular, the experiment was repeated 20 times to test the compression and recovery features.

#### Swelling Property

The swelling ratio (SR) was measured by a previously reported approach (Huang et al., 2019). The resulting CCS/CHOs, CCS/CNC-Ag, and CCS/CHO-Ag hydrogels (*n* = 3) were soaked in PBS solution (pH = 7.4) for 24 h to swell completely.

#### Adhesion Performance

Tensile adhesion tests were conducted to investigate the adhesive capacities of the hydrogels (Huang et al., 2019). In brief, the samples (25 mm × 20 mm × 2.5 mm) were pulled to fracture at a rate of 5 mm/min by the mechanical testing machine equipped with a 50 N load. The adhesion strength was calculated as the maximum load divided by contact area. All experiments were repeated at least three times.

### Ag^+^ Release Analysis and Antibacterial Activity *In Vitro*

To evaluate the release of Ag^+^ and Ag loading efficiency calculated from the mass percentage of the loaded Ag to the feeding Ag, atomic absorption spectroscopy (AAS; PE, AA700, United States) was performed. Before the examination, the release profiles of silver from the hydrogels were characterized *in vitro* by the all-change method in the PBS solution (pH 7.4). Here, at 37°C, CCS/CNC-Ag and CCS/CCHO-Ag (25 µg/sample) hydrogels were immersed in the PBS solution (5 ml) and shaken (100 rpm). At predetermined intervals, the released PBS was collected and replaced by fresh PBS. The amount of released Ag was determined using an AAS instrument, and the release profiles were obtained. The tests were repeated three times.

The germ-killing ability of the hydrogels was evaluated by turbidimetry measurements using *Staphylococcus epidermidis* (*S. epidermidis*, ATCC6538, Gram-positive organism) and *Escherichia coli* (*E. coli*, ATCC8739, Gram-negative organism) (Huang et al., 2019). By extension, the sterilized samples were placed into tubes, and then 5 ml of a bacterial suspension (1 × 10^4^ CFU/ml) was added to completely immerse the hydrogels, which were served as the experimental groups. However, the control group only contained the same volume of bacterial suspension in the test tube. Subsequently, all groups were incubated in the bacterial incubator at 37°C and 5% CO_2._ After incubating for 1, 4, 7, and 10 days, respectively, 500 μl of the bacterial suspension was taken to measure through turbidimetry at 570 nm. In addition, the antibacterial rate (AR) can be calculated using the following formula (Annabi et al., 2017):

$$AR=\frac{I_{1}-I_{2}}{I_{1}}\;\times \;100\%,$$

where *I*_1_ represents the OD value of the control group, while *I*_2_ for the experimental groups.

### *In Vitro* Cell Experiment

Human umbilical vein endothelial cells (HUVECs) were used to evaluate the biocompatibility of different hydrogels. Specifically, sterilized cylindrical hydrogels about 7 mm in diameter and 1 mm in deep were immersed in HUVECs with a density of 2 × 10^4^ cells/well. The cells were cultured in RPMI 1640 medium (Gibco) containing 10% FBS and 1% penicillin/streptomycin (P/S, Gibco). On days 1, 3, and 5, the cell proliferation and survival were assessed by using the CCK-8 assay and LIVE/DEAD® Viability/Cytotoxicity Kit. In brief, the hydrogel films of 1.5 mm thickness were fabricated and cut into 5-mm-diameter disks, and then these hydrogel samples were sterilized by alcohol immersion and UV irradiation process. The HUVECs were seeded into the 96-well plate (10,000 cells per well) and cultured for overnight. The hydrogel disks were introduced into the wells, then incubated at 37°C for 1, 3, and 5 days. At each time point, 10 µl Cell counting Kit-8 (CCK-8, Jiancheng, Nanjing, China) was added into each well, followed by incubation at 37°C for 2 h, then the absorbance value at 590 nm was measured by using a microplate reader. Meanwhile, the cells after co-cultured with hydrogel films for 5 days were washed with PBS twice before Live/Dead staining. After that, 200 µl of acridine orange/ethidium bromide (AO/EB, Beyotime, Shanghai, China) was added into each well and incubated in the dark at room temperature for 5 min, then observed by an inverted fluorescence microscope. And then, the living and dead cells were counted using the ImageJ software (V1.8.0.112).

To evaluate the migration ability, the cell transwell experiments of HUVECs were conducted on the surface of hydrogels samples. In brief, the treated cells with a density of 5 × 10^5^ cells/well were collected and resuspended in the surface of a transwell insert with the addition of 200 μl of culture medium with 1% FBS. After 48-h incubation with the addition of a full culture medium in the lower chamber, the resultant cells were fixed with 4% polyoxymethylene and stained with 0.5% crystal violet. Then five fields were chosen to photograph and count. In addition, the neovascularization capacity of the extracellular proteins was evaluated by immunofluorescence staining. In brief, the hydrogel samples were washed three times with PBS and then fixed with 4% polyformaldehyde for another 15 min. After incubating with anti-ANG (ab2831, Abcam) antibodies for 12 h, the cells were stained by DAPI solution for 15 min. The hydrogels were rinsed with PBS four times at 5 min per wash and were observed and photographed *via* CLSM.

### *In Vivo* Animal Experiments

The whole animal experiments were approved by the Animal Ethical Committee of Guangzhou Sport University SD rats. Before preparing the hydrogel, all precursors sterilized by filtration (0.22-μm filter, Millipore), and the hydrogel dressings were fabricated as described before in the section of hydrogel preparation. To establish the full-thickness skin defect model, streptozotocin (STZ, 60 μg/g) solution was injected into Sprague Dawley rats (male, 180–200 g) through their tail vein. The criteria for successful establishment were defined as fasting blood glucose above 16.7 mmol/L. Then the full thickness skin round wounds with 8 mm diameter were created by a needle biopsy. After the removal of wound skin, the obtained CCS/CCHOs and CCS/CCHO-Ag hydrogels were injected into the wound area until they completely covered the entire wound, and then the petrolatum gauze and elastic bandage with surgical suture were used to strongly fix the dressing in the wound area. For the control group, the control wounds were added to 40 μL of PBS and were additionally covered with a 4-cm × 10-cm piece of Tegaderm TM dressing (3M, St. Paul, MN, United States). Wound conditions of rats were observed and recorded on 0, 5, 10, and 15 days, respectively. At the same time, the wound area was measured *via* ImageJ software. Wound contraction (%) was calculated using the formula below: Wound contraction= [area (0 day)-area (n day))/(area (0 day)] ×100%, where “n” represents the day, such as 5th, 10th, and 15th days. All results were analyzed by using the one-way ANOVA test.

### Histological Analysis

Wound tissues were collected and fixed with 4% paraformaldehyde solution on days 5, 10, and 15. The harvested samples were stained with hematoxylin and eosin (H&E), and immunohistochemical staining of IL-1β and IL-6 was performed using a standard protocol on adjacent sections for confirming the pro-inﬂammatory cytokine levels after the hydrogels treatment *in vivo*. In addition, the immunofluorescence staining of CD31 was conducted according to standard protocol (Zhang et al., 2019). All the slices were observed under a microscope (IX53, Olympus, Japan).

### Statistical Analysis

All experiments were repeated three times, and data were shown as mean ± SD. One-way ANOVA, following Tukey’s test, was carried out for statistical analysis by GraphPad Prism software. In particular, we defined *p* < 0.05 as statistically significant (*p < 0.05, **p < 0.01, and ***p < 0.001).

## Results and Discussion

### Synthesis and Characterization of CCHOs

**FIGURE 1:**
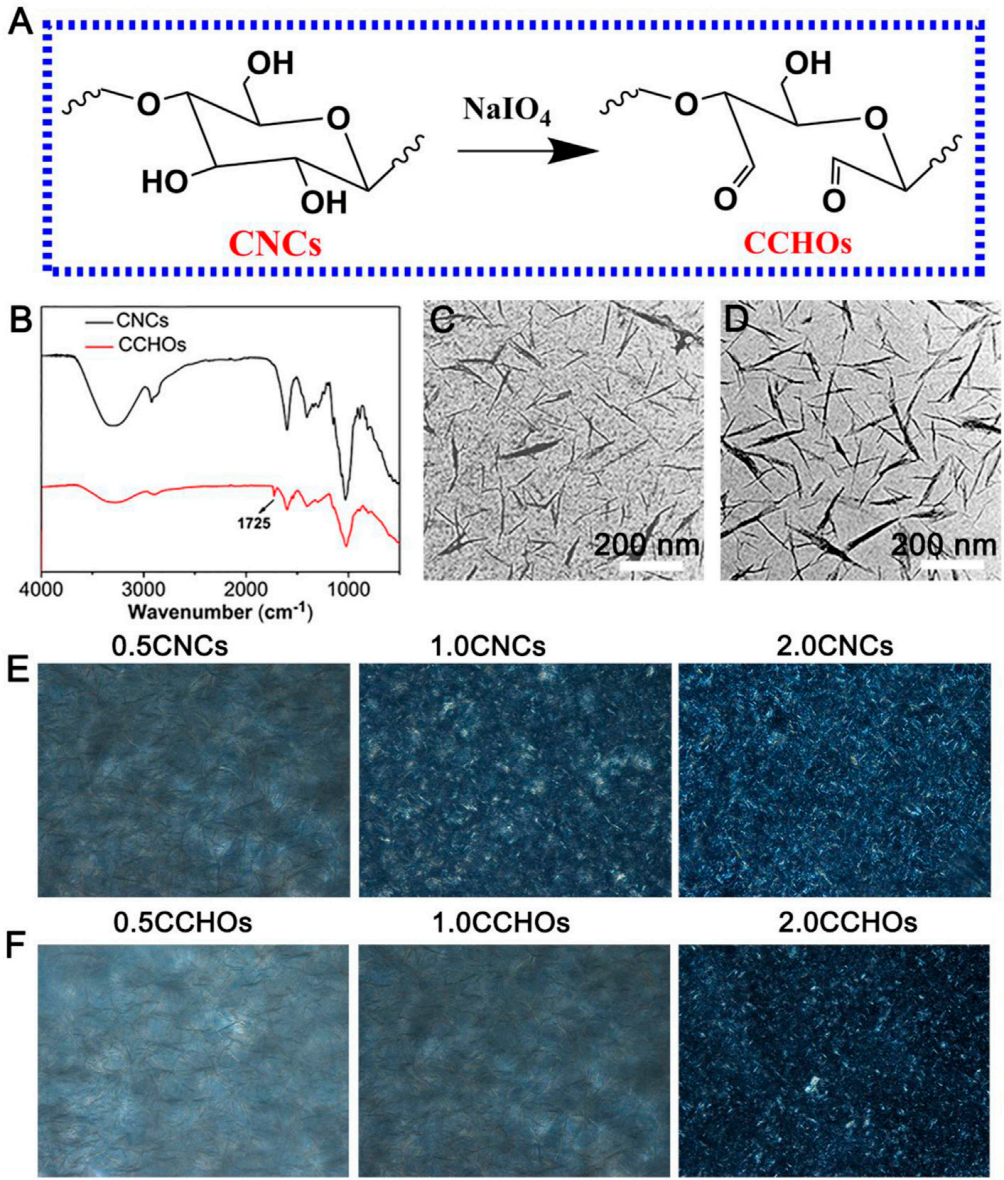
**(A)** Schematic of synthesis of CCHOs. **(B)** FTIR spectra of the CNCs and CCHOs. TEM image of CNCs **(C)** and CCHOs **(D)**. The corresponding POM images of CNC **(E)** and **(F)** CCHO whisker aqueous suspensions with 0.5, 1, and 2% concentrations.

As a specific marker of biofilms, CNC liquid crystal (LC) has been known for its positive effects on cell affinity, migration, and proliferation (Zhang et al., 2020; Xie et al., 2020). Here, the CNCs were modified with NaIO_4_ (Figure 1A). In order to validate the successful surface-modified aldehyde groups of CNCs, we carried out the attenuated total reﬂection Fourier transform infrared (ATR-FTIR) (Figure 1B). When compared with the spectrum of CNCs, CCHOs showed a new peak that appeared at 1725 cm^−1^, which can be explained as the stretching vibration of C=O of the aldehyde group on its surface after oxidation (Chen et al., 2021). The content of aldehyde groups of the CCHOs was evaluated to be 7.30 mmol/g using a NaOH titration approach. The morphology of the CCHOs was observed by TEM. It can be seen that the CNCs and CCHOs exhibited a needle-like morphology without noticeable change after modification (Figures 1C,D). The results suggested that aldehyde modification did not play a significant role in the average length and diameter of the whiskers. According to the POM observation results, both the CNC and CCHO aqueous suspensions showed the typical cholesteric texture at 37°C from anisotropic materials. As the content increased, the domains of the LC phase gradually increased, and the field of view under POM gradually became brighter (Figures 1E,F), confirming that the modification of CNCs did not influence on its LC property.

### Fabrication of the CCS/CCHO-Ag Hydrogels

**FIGURE 2:**
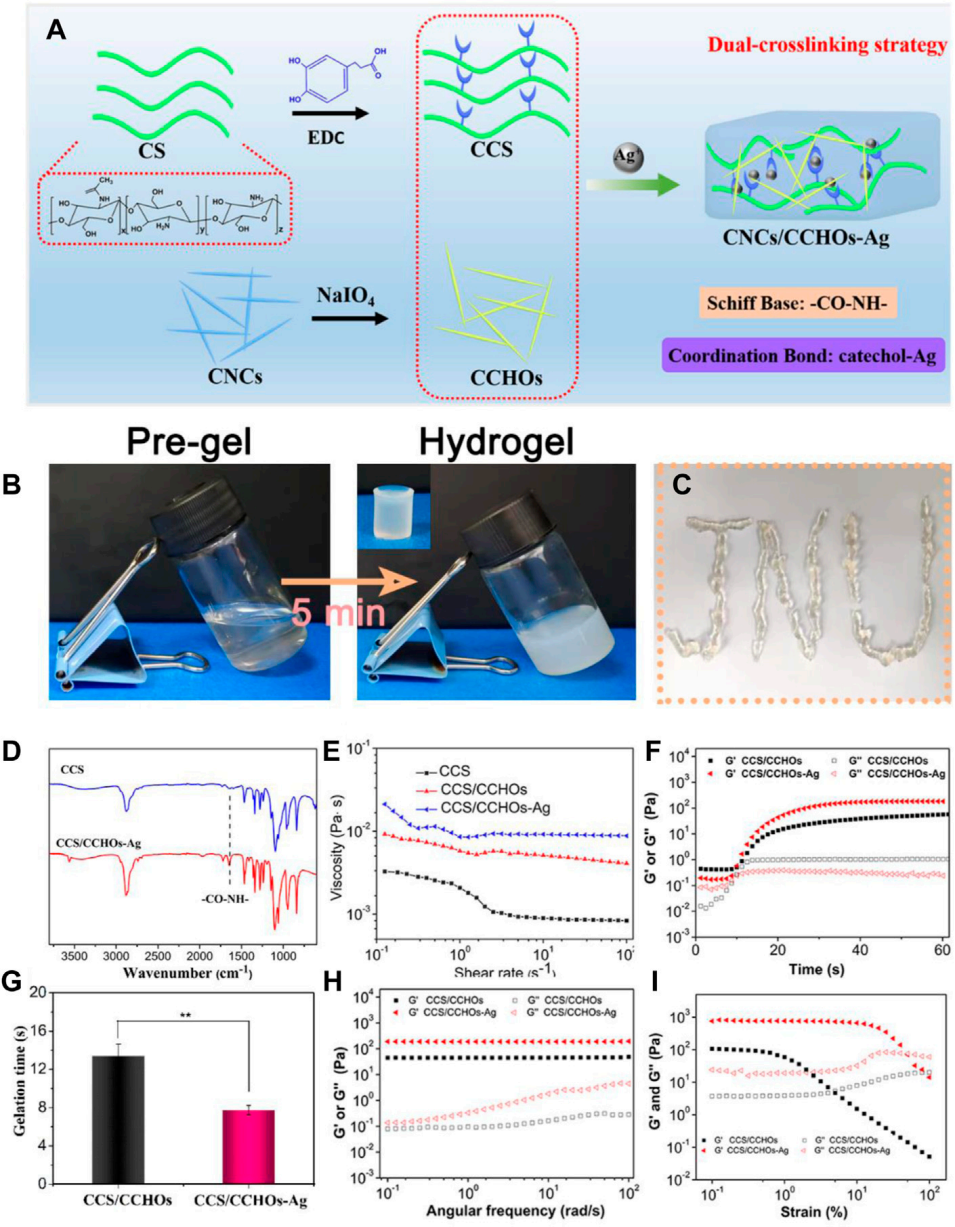
**(A)** Scheme of CCS/CHO-Ag hydrogel synthesis by a dual-cross-linking strategy. **(B)** Optical images of hydrogels *via* a tube inverted test. **(C)** Injection process of the resultant hydrogel. **(D)** FTIR spectra showed the formation of the CCS/CHO-Ag hydrogel. The rheological results of obtained hydrogels: **(E)** Viscosity and viscoelasticity of CCS, CCS/CCHOs, and CCS/CCHO-Ag whisker aqueous suspensions; **(F)** time sweeping of storage modulus (*G*′) and loss modulus (*G*″); **(G)** gelation time obtained by time sweeping test; **(H)** frequency-dependent (at a strain of 1%) and **(I)** strain-dependent (ω = 6.28 rad/s) oscillatory shear.

Figure 2A shows the synthesis pathways of CCS/CCHO-Ag nanocomposite hydrogel and its application to wound healing. In this study, the modified CS with 3, 4-dihydroxy hydrocinnamic acid (CCS) is the matrix material of the hydrogel system. Based on the activation of EDC, the carboxylic group of hydrocaffeic acid was reacted with an amino group in CS to produce an amide bond. The efficiency of catechol grafting onto the CS was about 23.1%, which was verified by ^1^H NMR and UV-vis analysis (Supplementary Figure S1, Supporting Information). Then CS was mixed with CCHOs, followed by incorporation of Ag^+^ to form CCS/CCHO-Ag hydrogel based on Schiff base bonds (amino group of CCS and aldehyde group of CCHOs) and coordinate bonds (Ag and catechol group of CCS). The tube inverted test ensured the successful formation of the dual-cross-linking hydrogel. Since the hydrogel is composed of the dynamically cross-linked network, the resulting hydrogel showed good injectability (Figure 1C). Also, its chemical structure was further studied using FTIR. According to the FTIR spectra (Figure 2D), a strong peak at 1637 cm^−1^ was observed, indicating the formation of imine bonds (Tang et al., 2021). Meanwhile, the CCS/CHO-Ag hydrogel specimen showed a new band at 1350 cm^−1^, which may be seen as the interaction between Ag and catechol group. These results demonstrated the successful formation of dual-cross-linking hydrogel based on Schiff base bonds and coordinate bonds between the Ag and catechol groups.

In order to further reveal the gelation mechanism of CCS/CCHO-Ag, a series of rheological tests of composite hydrogels were conducted. First, the shear viscosity of the different pre-gel solutions was measured by a rotating rheometer. As shown in Figure 2E, the shear viscosity of CCS/CCHO solution exhibited an obvious increase compared with pure CCS solution with a shear rate of 0.1 s^−1^, with the addition of Ag, the viscosity continues to increase. This may result from a loose network formed *via* the interaction between the catechol group and Ag, which can be destroyed as the improvement of shear rate (Huang et al., 2017). Next, rheological measurements were conducted using different sweeps (time, frequency, and strain) during the gelation process. The *G*′ value gradually increased from 0 to 60 s and then intersected the *G*″ value, indicating the transition point of sol-gelation (Figure 2F). Due to the dual-cross-linking mechanism, the gelatin time of the CCS/CCHO-Ag hydrogel group was shorter than the pure CCS/CCHOs hydrogel group (Figure 2G) and possessed higher equilibrium modulus (*G*_*e*_′). Figure 2H demonstrates the frequency sweep of hydrogels samples. The *G*′ value maintained constant during the whole frequency sweep process, which meant the characteristics of a well-developed cross-linked network (Huang et al., 2019). In addition, strain-dependent oscillatory rheology of the CCS/CCHO-Ag hydrogels showed a stress relaxation when the strain exceeded 50%, which suggested that the processing range was wide because of their shear thinning property (Figure 2I).

### Scanning Electron Microscope Observation, Mechanical Performance, Swelling Behaviors, Adhesive Properties, and Morphology of the Hydrogels

**FIGURE 3:**
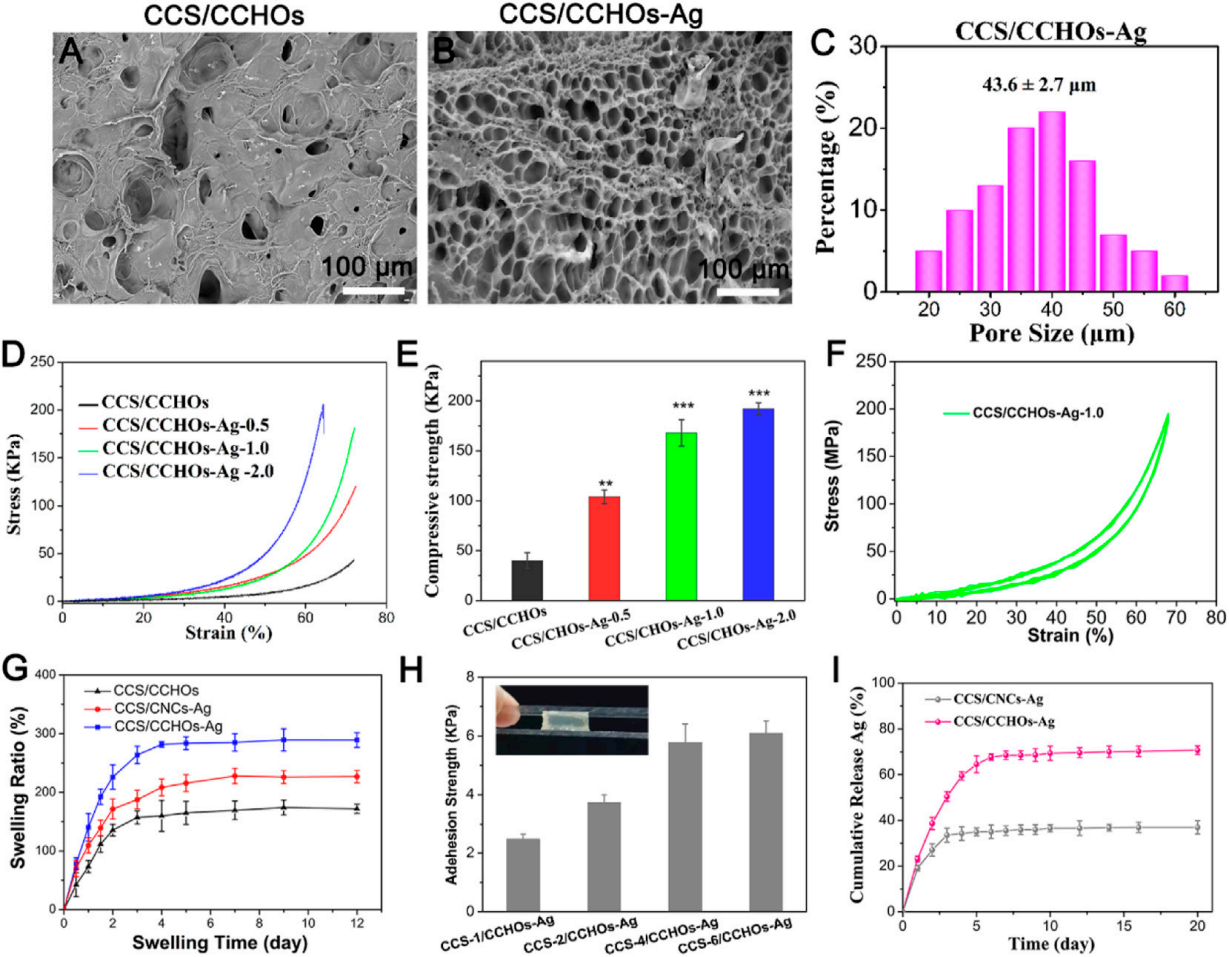
SEM images of CCS/CHOs **(A)** and CCS/CHOs–Ag **(B)**, and statistical pore size of CCS/CHO-Ag hydrogel **(C)**. **(D)** Compressive stress–strain curves of Gel-DA/DOHA/DMON@Fe hydrogels with a molar ratio of 0.5, 1.0, and 2.0 for -CHO/-NH_2_ and **(E)** its corresponding results of mechanical strength and Yong’s modulus. **(F)** In total, 20 successive compressive loading-unloading cycles for hydrogel with a strain of 70%. **(G)** Swelling rate. **(H)** Adhesion strength of hydrogels with different CCS content adhered to porcine skin. **(I)** *In vitro* release profiles of Ag^+^ from the hydrogel scaffolds.

As can be seen from Figure 3A, scanning electron microscope (SEM) images demonstrated that the collapsed hole structure was observed in CCS/CHOs hydrogel owing to unstable cross-linked structure easy to change through rapid lyophilization, while the CCS/CHO-Ag displayed open porous structure, with the average diameter around 30–50 µm (Figures 3C,D). The introduction of Ag^+^ promoted the formation of uniform networks *via* a dual-cross-linking strategy. The interconnected porous structure is beneficial to the transmission of nutrients for cells (Jin et al., 2020). A typical compressive test was conducted to assess the mechanical behavior of the CCS/CHO-Ag scaffold, and the mechanical properties of the above hydrogels were illustrated (Figures 3D,E). The compressive strength and Young’s modulus of the CCS/CCHO-Ag hydrogels were significantly higher than those of the CCS/CCHOs hydrogel because of the formation of Ag-catechol coordination that strengthened the microfibrils and improved the overall strength of the composite scaffolds. The compressive stress of CCS/CHOs dual-cross-linking hydrogel containing 0.5, 1.0, and 2.0 wt% of Ag increased to 107, 163, and 183 KPa, respectively, and its corresponding modulus values increased to 275, 432, 258.6, and 603 KPa, respectively, which showed significant improvement compared with the CCS/CHOs sample. It is noted that CCS/CCHO-Ag-2.0 samples showed reduced ductility, which might be possible that in the presence of excessively high concentration CCHOs, the dual-cross-linking hydrogel may lead to weak energy dissipation and small stress tolerance of nanoparticles to deformation (Han et al., 2017). The mechanical performance of obtained CCS/CCHO-Ag system is superior to previously reported histatin 1-modified thiolated chitosan dressings (Lin et al., 2020). In addition, the CCS/CCHO-Ag-1.0 was chosen as the sample for the following experiments. As expected, 20 cycles of loading–unloading compression tests were operated using a strain of 70% (Figure 3F). The formed CCS/CCHO-Ag hydrogel could withstand 70% of the strain, indicating that the hydrogel showed excellent resilience and toughness properties.

Considering that hydrogel wound dressings should have a certain degree of water absorption capacity for the clearance of exudates during the process of wound healing, swelling behaviors of the hydrogel are needed to be evaluated (Lin et al., 2020). The swelling ratio of the hydrogels was also consistent with the above SEM results. Figure 3G shows that the dual-cross-linking CCS/CCHO-Ag hydrogels possessed a higher swelling rate than pure CCS/CCHOs sample, and CCS/CCHO-Ag-1.0 reached 274.6% after 12 h, indicating the dual cross-linking hydrogel possess desirable water absorption capacity owing to formation of large interpenetrating pores for absorbing more water molecule, which is almost 2 times that of previously reported CS double network hydrogel (Huang et al., 2019). A good wound dressing required not only to accelerate skin regeneration but also firmly attach to surrounding tissues, so we evaluated the adherence of hydrogels *via* the lap shear test using pigskin (Figure 3H). The adhesion abilities improved with the increase of the amount of CCS incorporated, and the long-term adhesion ability of CCS-6/CCHO-Ag was further reached to 5.8 KPa surprisingly, most likely from the enhancement of catechol chemistry–based hydrogel with long-term adhesiveness (Gan et al., 2019).

### Antibacterial Activities of Ag-Loaded Hydrogels

**FIGURE 4:**
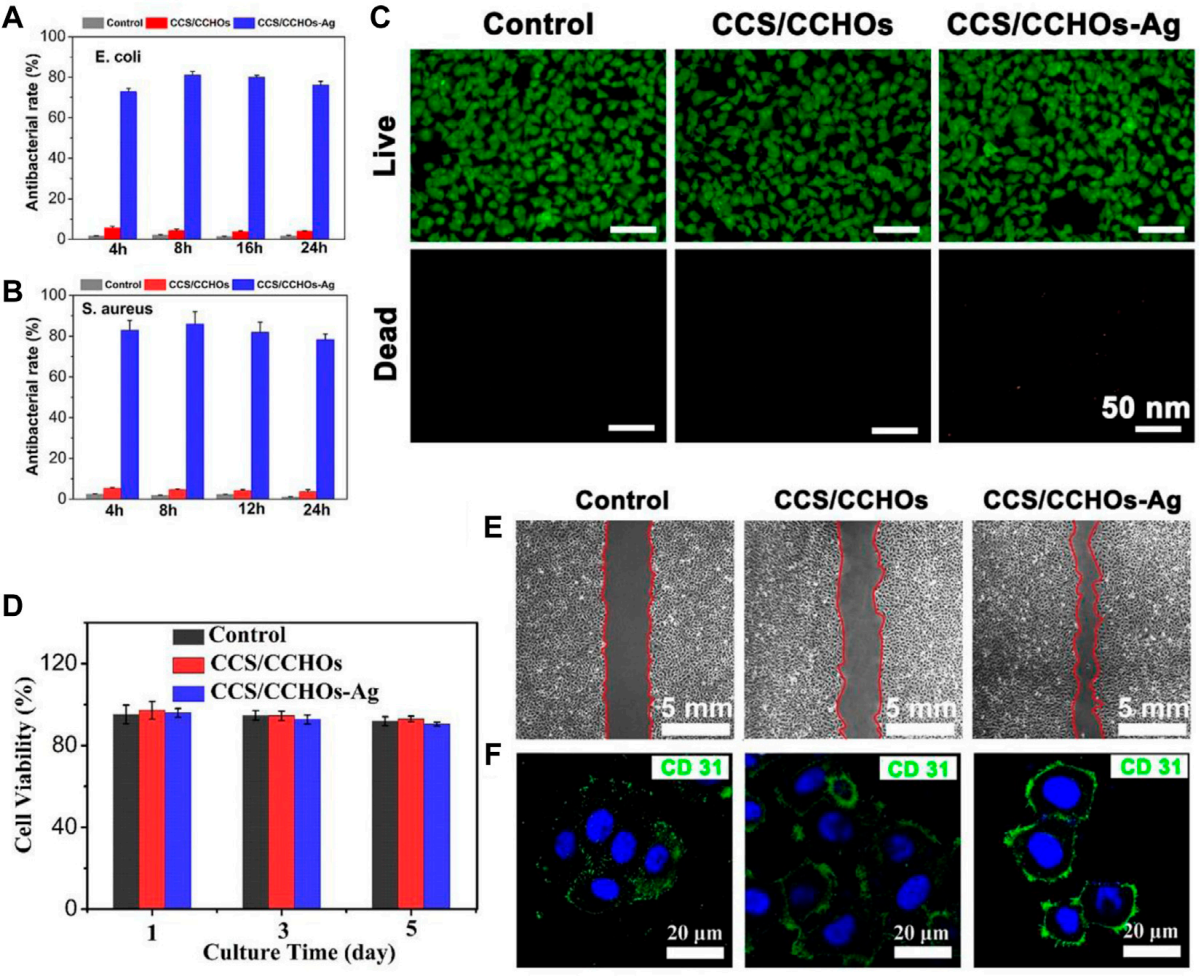
Antibacterial rate of corresponding samples against *E. coli* **(A)** and **(B)** *S. aureus* after co-culturing for 4, 8, 16, and 24 h. **(C)** Live/Dead staining of HUVECs cultured on hydrogel scaffolds for 5 days. **(D)** CCK-8 assay of the proliferation of cells. **(E)** Transwell migration assays of HUVECs. **(F)** Fluorescence staining for CD 31 (green) and nucleus (blue) in HUVECs cultured with hydrogel sample.

The sustained antibacterial activity to fight infection has a significant meaning in wound healing (Garakani et al., 2020). Silver has potential biosafety risks despite its broad-spectrum antibacterial activity (Yang et al., 2021). Thus, the Ag-loaded hydrogels possess controlled release property. As shown in Figure 3I, the CCS/CCHO-Ag specimen presented a faster release rate during the first three days and slower subsequent release of Ag within 7 days. The denser coordination between Ag and CCS of hydrogels structure can be regarded as a barrier to prevent the Ag release rate and lead to more stability and sustained release in 10 days compared to CCS/CNC-Ag group. It is note that the amounts of Ag release from CCS/CCHO-Ag was much higher than that from CCS/CNC-Ag group because of the higher Ag loading efficiency (83.6 ± 3.7 vs. 39.8% ± 4.1%) after incubated for 21 days. In addition, the amounts of Ag^+^ released from the CCS/CCHO-Ag hydrogels were 0.4877 mg/L after 7 days of release. This low concentration of Ag^+^ ions is safe for cells and tissues (Basha et al., 2020). These results suggests that the CCS/CCHO-Ag hydrogels are capable of providing sustained release of active substances and contributing to protect the wounds from infection for a long time. Subsequently, the evaluation of the antimicrobial activity of hydrogels was based on the turbidimetry method, and the Ag-loaded hydrogels exhibited more remarkably enhanced antimicrobial features against both *E. coli* and *S. aureus* when compared with CCS/CCHOs samples (Figures 4A,B). More importantly, the antibacterial rate was consistently above 80% during incubation at 4, 8, 16, and 24 h, which is ascribed to the sustained release of Ag. All the results suggested that the hydrogel had the ability to minimize the growth of both G− and G+ bacteria.

### Cytocompatibility and Angiogenesis Assay *In Vitro*

Furthermore, the biocompatibility of the fabricated scaffolds was tested *via* cytotoxicity test, including the examination of cell counting and Live/Dead staining. The HUVECs were co-cultured with CCS/CCHOs and CCS/CCHO-Ag, and the survival rate of cells in both groups was over 90% at 5 days (Figure 4D), which was consistent with the results of Live/Dead staining (Figure 4C), suggesting that the addition of Ag exhibited good biocompatibility without obvious detrimental effects on cell growth. Apart from that, angiogenic activity plays another important role in accelerating wound healing (Li et al., 2019; Zhang et al., 2017). Also, the transwell migration behavior of HUVECs cultivated on the surface of various hydrogels was conducted. As shown in Figure 4E, no significant increase in migration was shown in the control group. The significant cell migration appeared in CCS/CCHOs and CCS/CCHO-Ag, and the maximal rate of migration was observed in the dual-cross-linking scaffold. Meanwhile, CD 31 (green) immunofluorescence staining was further performed to study the revascularization ability of the endothelial cells (Figure 4F). According to the highest expression of CD 31 displayed in the CCS/CCHO-Ag hydrogel group, undoubtedly it had the best revascularization ability. Firstly, previous studies have revealed that CNCs LC state is an important basis for the induction of angiogenesis, thanks to its oriented structure, thus leading to the promotion of cell adhesion and proliferation (Zhai et al., 2012; Liu et al., 2020). Secondly, dual-cross-linking scaffolds co-regulate the cell–matrix relationship by stimulating anisotropic viscoelastic and biochemical interactions between receptors and ligands, providing an appropriate substrate for three-dimensional (3D) cell growth (Lin et al., 2019). Taken together, the composite system containing CNCs LC exhibited synergistic effects on promoting the formation of vascularization, which could be seen as an excellent skin dressing.

### *In Vivo* Evaluation of Wound Healing

**FIGURE 5:**
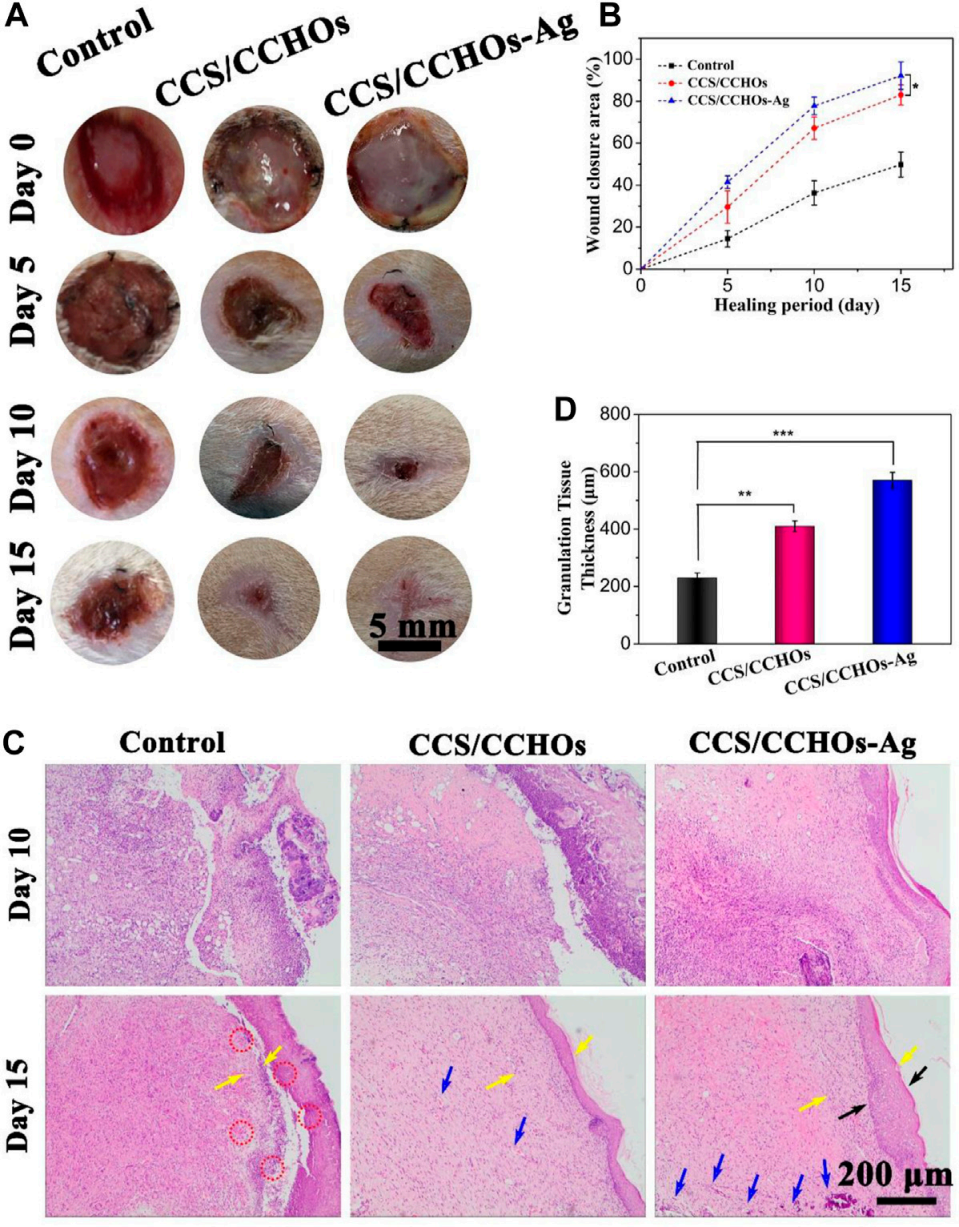
**(A)** Optical images of wounds untreated or treated with CSS/CCHO and CCS/CCHO-Ag dressings on days 0, 5,10, and 15. **(B)** Corresponding healing rate of wounds. **(C)** Tissue slices stained with hemotoxylin and eosin (H&E) from different groups on days 10 and 15. **(D)** Quantitative analysis of the granulation tissue thicknesses (as pointed by the yellow arrows) using ImageJ.

Inspiring the excellent angiogenesis and antibacterial activity of resulting hydrogel *in vitro*, a full-thickness skin defect model was created to evaluate the effect of different hydrogels on promoting wound healing. All mice were divided into three groups totally: no treatment group (the control group), CCS/CCHOs hydrogel treatment group, and Ag-incorporated CCS/CCHOs treatment group. In view of capable of sustained Ag release from CCS/CCHO-Ag, we reasonably speculate that the our hydrogel dressing has a long-lasting therapeutic effect on wound healing, therefore the full thickness skin round wounds covered by hydrogels without additional dressing treatment. As shown in Figure 5A, on days 5 and 10, yellow pus was observed on the bright red wound surface in the control group, suggesting that the injured site was going through inflammation. However, hydrogel dressings groups showed more tidy wound areas with faster contraction rates, perhaps because of a moist environment maintained by the wound dressing (Huang et al., 2019). In particular, the CCS/CCHO-Ag hydrogel group covered the wound completely with the newly-formed epidermis, showing the fastest wound healing rate. On day 15, the wound closure area of CCS/CCHOs and CCS/CCHO-Ag group were 92.5 ± 6.5% and 77.5 ± 4.1%, respectively, which clearly superior than control group (41.7 ± 3.1%). It is noted that the efficiency of wound healing between CCS/CCHOs and CCS/CCHO-Ag displayed statistical significance (*p < 0.05) by one-way ANOVA tests with a Tukey model, indicating wound healing got better results from the incorporated Ag composite system by inhibiting infection to reduce inflammation.

To evaluate the quality of wound healing, histological analysis was conducted on days 10 and 15. On the 10th day, there were fewer inﬂammatory cells (as pointed by the red circle) but more fibroblast cells in the CCS/CCHOs and CCS/CCHO-Ag hydrogel groups than the control group (Figure 5C), thanks to the sustained release of Ag^+^ from CCS/CCHO-Ag system, which could significantly control inflammation. In addition, more blood vessels (as pointed by the blue circle) in these two groups could be attributed to the LC component that enhanced angiogenesis and accelerated wound regeneration as well (Xie et al., 2020; Zhan et al., 2021). After 15 days, a large area of immature granulation tissues were observed in the control and CCS/CCHOs hydrogel groups, while normal epithelium and almost completely regenerated dermal tissue were shown in the CCS/CCHO-Ag group. In addition, the dual-cross-linking hydrogel treated group showed the thickest granulation tissue and formed normal epidermal–dermal layer structure (as pointed by the black arrows), indicating this hydrogel achieved the best results concerning wound healing among these three groups (Figure 5D). In brief, the prepared CCS/CCHO-Ag hydrogel was the optimal wound dressing because it could promote skin regeneration.

### Anti-inflammatory and Vascularization Effect of Hydrogels *In Vivo*

**FIGURE 6:**
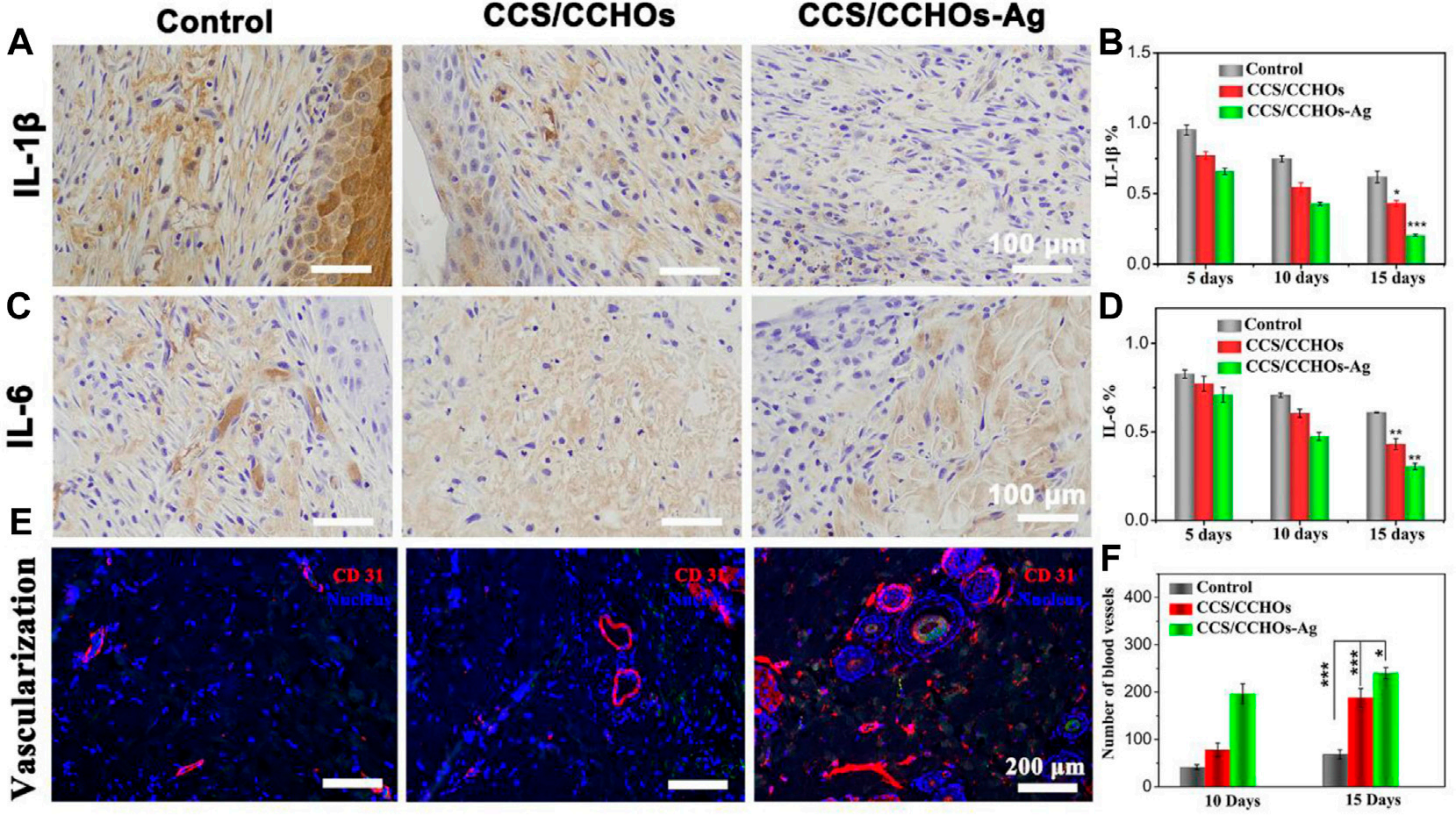
Immunohistochemical staining of pro-inﬂammatory factors: **(A)** IL-1β and **(C)** IL-6 after different treatments for 15 days, and **(B,D)** its corresponding results of semi-quantitative analysis of immunohistochemical staining. **(E)** Immunofluorescence staining of CD31 for blood vessel formation *in vivo*, and **(C)** quantitative counts of newly formed blood vessels per mm^2^ at 15 days.

The inflammatory reaction is so severe in some infections that the wound repair can be delayed (Barkhordari et al., 2014; Zhao et al., 2017). IL-1β and IL-6, two typical inflammatory cytokines, are usually used to assess the early and middle stages of inflammatory response in the process of wound healing, respectively (Liu et al., 2018). Figures 6A–D show the lower expression of IL-1β and IL-6 in CCS/CCHO-Ag and CCS/CCHOs hydrogels rather than the control group, which represented less inflammatory response. Specifically, the highest level of inflammatory response among the three groups was the control group, while the lowest was the CCS/CCHO-Ag group. On the 15th day, the expressions of IL-1β and IL-6 presented a relatively higher level in the CCS/CCHOs groups, while the inflammatory responses of the CCS/CCHO-Ag group almost disappear, implying that the Ag-loaded hydrogels were able to effectively prevent infection and to promote wound repair.

Next, we explored the influence of CCS/CCHO-Ag on the formation of blood vessels *in vivo*. The blood vessel density was evaluated by immunofluorescence staining of CD31 that is mainly used to demonstrate the presence of vascular endothelium. CCS/CCHOs and CCS/CCHO-Ag group showed the property of increasing vessel numbers because the introduction of CNCs LC triggered the angiogenic process *in vivo* (Figure 6E). Also, quantification analysis reveals a significantly higher number of blood vessels in the CCS/CCHO-Ag than the control group (Figure 6F), suggesting the good anti-inflammatory ability in Ag-loaded hydrogels could also promote the formation of blood supply (Eming et al., 2007). Therefore, the prepared dual-cross-linking hydrogel system plays a combined role in regulating the inflammatory microenvironment and promoting angiogenesis, showing synergistic effects triggered skin repair.

## Conclusion

To sum up, a multifunctional skin wound dressing with self-remodeling and biocompatible property was prepared, and its repairing potential in the full-thickness wound was also well-demonstrated. The fabrication of CCS/CCHO-Ag hydrogel was based on the double cross-linking strategy using the dynamic Schiff base and catechol–Ag coordination, which endowed the hydrogel with superior mechanical strength, strong tissue adhesion, perfect antibacterial activity, and good biocompatibility property. The LC-mimetic hydrogel showed a strong synergistic effect by the paracrine mechanism to significantly improve the migration and to stimulate angiogenesis of HUVECs *in vitro*. Also, the animal experiments demonstrated that CCS/CCHO-Ag scaffold had remarkable antibacterial activity and anti-inflammatory effects due to the one-demand release of the Ag. At the same time, the scaffold provided an anisotropic viscoelastic microenvironment that could promote neovascularization, jointly accelerating the wound healing process. In conclusion, the aforementioned satisfactory results proved that the multifunctional hydrogel is expected to be used for wound healing and provoke more interest in stimulus-responsive wound dressings.

## Data Availability

The original contributions presented in the study are included in the article/Supplementary Material, further inquiries can be directed to the corresponding authors.
