# Supplementary material for: Antibacterial Cellulose Nanocrystal-Incorporated Hydrogels With Satisfactory Vascularization for Enhancing Skin Regeneration
Source: Front Bioeng Biotechnol. 2022 Apr 26;10:876936. doi: 10.3389/fbioe.2022.876936 (PMC9086275; doi:10.3389/fbioe.2022.876936)
Supplement: Supplementary file 1 [file Image1.pdf]

Supplementary material

Antibacterial cellulose nanocrystals incorporated-hydrogels with satisfactory vascularization for enhancing skin regeneration

Haibin Lu [1, 2], Xiaoling Li [1], Mu Zhang [1], Changpeng Xu [3], Wenqiang Li [4, *], Lei Wan [1, *]

[1] Stomatological Hospital, Southern Medical University, Guangzhou 510280, China.

[2] Shunde Hospital, Southern Medical University (The First People's Hospital of Shunde), Foshan 528308, China.

[3] Engineering Technology Research Center for Sports Assistive Devices of Guangdong, Guangzhou Sport University, Guangzhou China.

[4] Guangzhou Sport University, Guangzhou 510500, China.

*** Corresponding author:**

Wenqiang Li gztylwq@foxmail.com;

Lei Wan wanleilucky@126.com.

1.1 Determination of aldehyde group content (AGC)

The AGC of CCHOs was determined through a NaOH titration method referred to previous studies[29]. Briefly, The pH value of a known amount of CCHOs suspension was adjusted to 5 with 0.1 N HCl solution. Then, 5% (w/w) hydroxylamine hydrochloride ($H_2NOH•HCl$) solution at pH 5 was added into CCHOs suspension. The mixture was stirred for 3 h in a hot water bath at 40°C. The released HCl was titrated with 0.1 N NaOH solution to keep the pH of the mixture at 5; thus, the 0.1 N NaOH solution was dropped continually till no decrease of pH was observed. Here, the consumption of 0.1 N NaOH solution in liters was recorded as V_2. The same concentration of CCHOs suspension at pH 5 was used as a blank, and its consumption of the NaOH in liters was recorded as V_1. The weight of the CCHOs sample (W, g) was measured after it was completely dry. Thus, AGC (mmol/g) in CCHOs could be calculated by following Eq: AGC = $(V_1 - V_2) \times 0.1/W$. Finally, the AGC on the CCHOs was related to the reaction time which was measured to be 7.30 mmol/g for 12 h.

1.2 Characterization of catechol-modified chitosan (CCS)

To confirm the conjugation of the catechol group, the synthesized CCS conjugate was dissolved in deuterium oxide and then analyzed using proton nuclear magnetic resonance (^{1}HNMR) spectroscopy at 300 MHz. In addition, the CCS conjugate (1 mg/mL) was dissolved in acidic PBS (pH 5) and measured the absorbance at 280 nm ultraviolet−visible (UV−vis) spectros-copy (JASCO Corporation, Tokyo, Japan) to verify the presence of catechol groups.

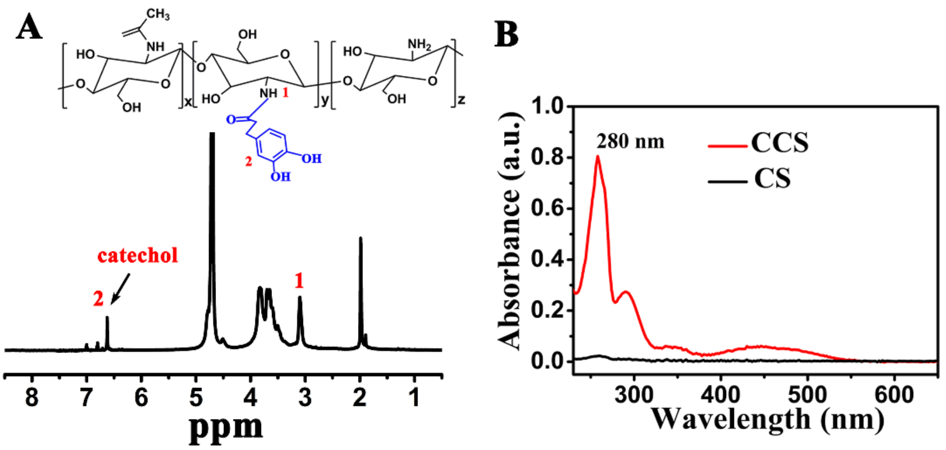

Figure S1. (A) ^{1}H NMR spectra of CCS, (B) UV-Vis spectra of CCS and unmodified

CS.
